# Determinants of health-related quality of life among patients with systemic lupus erythematosus in Hanoi, Vietnam

**DOI:** 10.1186/s41927-023-00339-6

**Published:** 2023-06-22

**Authors:** Aya Mizukami, Minh Trang Trinh, Thi Phuong Hoang, Akira Shibanuma, Ken Ing Cherng Ong, Masamine Jimba

**Affiliations:** 1 Department of Community and Global Health, Graduate School of Medicine, The University of Tokyo, Bunkyo-ku, Tokyo, Japan; 2 National Hospital of Dermatology and Venerology, Hanoi, Vietnam

**Keywords:** SLE, HRQoL, Vietnam, Lower middle-income country

## Abstract

**Background:**

Systemic lupus erythematosus (SLE) is a chronic autoimmune disease which impacts patients’ lives. Many studies in high-income countries have focused on their health-related quality of life (HRQoL). However, evidence of awareness of SLE and HRQoL in low- and middle-income countries is lacking. Therefore, this study aimed to identify the determinants of HRQoL of SLE patients in Vietnam, a lower-middle income country.

**Methods:**

This cross-sectional study was conducted at the National Hospital of Dermatology and Venereology in 2019. A pre-tested structured questionnaire was used to collect data. It consisted of Short Form-36 to assess HRQoL which comprised physical and mental component summaries, Multidimensional Scale of Perceived Social Support, Satisfaction with Life Scale, and Mental Adjustment to SLE. Multiple linear regression was used to identify the determinants of HRQoL.

**Results:**

One hundred thirty four patients with SLE participated in this study. The majority of the patients were women (n = 126, 94.0%). The mean age of all participants was 37.9 years old (standard deviation [SD] 12.5). Of 134 participants, 104 (77.6%) were married. Older patients were more likely to have a lower score of mental component summary (B=-0.45, 95% CI -0.73, -0.17). Patients with more children were more likely to have a lower score of physical component summary (B=-5.14, 95% CI -9.27, -1.00). Patients who felt more helplessness or hopelessness were more likely to have lower scores of physical and mental component summaries (B=-1.85, 95% CI -2.80, -0.90; B=-1.69, 95% CI -2.57, -0.81). Also, patients who felt more anxious were more likely to have a lower score of mental component summary (B=-1.04, 95% CI -1.77, -0.32). Patients who were more satisfied with their lives were more likely to have higher scores of physical and mental component summaries (B = 1.07, 95% CI 0.50, 1.64; B = 1.08, 95% CI 0.55, 1.61).

**Conclusion:**

Factors associated with lower HRQoL in Vietnam were feelings of helplessness or hopelessness, and burdens of parenting roles. However, social support can contribute to a higher HRQoL, such as information support, self-support groups, and daycare services provided at the community level.

**Supplementary Information:**

The online version contains supplementary material available at 10.1186/s41927-023-00339-6.

## Background

Systemic lupus erythematosus (SLE) is a chronic autoimmune disease that causes organ and nervous system impairment and evokes skin manifestations. These impairments usually result in various combinations of symptoms that negatively affect patients’ health-related quality of life (HRQoL). SLE is prone to develop in women of reproductive age, and its prevalence among women is approximately 9 times higher than that among men [1]. Research on causes and treatments has been conducted and contributed to mortality and morbidity decline. However, patients’ HRQoL remains worse compared to the general population due to the variety of impairments and problems caused [2].

Most patients with SLE suffer from common physical and mental symptoms, such as fever, hair loss, depression, and invisible symptoms such as pain and fatigue. Although these chronic symptoms do not directly result in high mortality rates, they have a significant impact on the patients’ lives [3]. Invisible symptoms cause misunderstandings and a lack of support from family, friends, and even medical professionals [4, 5]. Visible symptoms such as skin manifestation and hair loss cause unfavorable appearances, which could result in low self-esteem in patients with SLE. Pregnancy is one of the biggest concerns among patients because it is known as a trigger for relapse, and most patients with SLE are women [6]. Unknown causes and treatments may lead to anxiety in patients regarding their future. Therefore, supports and understanding from others are important for the patients.

Social support plays a significant role in the daily lives of SLE patients. Social support involves physical or mental relationship transactions, either directly or indirectly, among individuals [7]. When family, friends, and significant others provide support and help enhance their self-esteem with their support, they can help patients maintain their health as well as disease recovery [8]. Hence, social support could bring many possible benefits to SLE patients’ lives, and it could assist them in being physically and mentally healthier amidst uncertainties of their situations. Better social support might facilitate a better HRQoL.

Mental adjustment can be defined as “the cognitive and behavioral responses the patients make to the diagnosis” [9]. In the case of patients with SLE, they might fail to manage their mental health and be inclined toward negative conceptions about their lives with inadequate social support [10]. Poor mental health managements and negative conceptions could lead to a low HRQoL. Assessing patients’ mental adjustment could provide insights into how they respond to the diagnosis and accommodate their daily life with SLE. Therefore, mental adjustment has been identified as an important factor affecting HRQoL [11].

Patients with SLE who have insufficient social support and poor coping mechanisms might not achieve what they plan for their life [4]. Satisfaction with life can be understood as contentment and is a part of subjective well-being connected with happiness and positive emotions [12]. Understanding their satisfaction with life is crucial for improving their HRQoL.

HRQoL is a multidimensional concept of how disease and treatment affect a patient’s sense of overall function and well-being [10] and includes 4 domains: physical, mental, emotional, and social functioning [13]. Assessing HRQoL helps us understand their situations holistically to improve their well-being [14]. In a systematic review from high-income countries in Europe, North America, and Australia, SLE affected the HRQoL of patients and their abilities to perform daily activities [15]. HRQoL of patients with SLE was consistently lower than the patients with other chronic diseases or the general population. Various determinants were reported to influence HRQoL adversely, such as (1) older age, (2) fatigue, and (3) the presence of co-morbid neurological or psychiatric disorders [15]. The European League against Rheumatism recommends evaluating the HRQoL at every hospital visit since HRQoL reflects the burden of SLE on individual patients [15]. However, the HRQoL of patients with SLE has not attracted much attention, especially in lower middle-income countries in Asia, such as Vietnam, where medical approaches tend to emphasize only treatments [16–18]. Therefore, studies on the factors associated with HRQoL among patients with SLE in Asian settings, especially in low- or middle- income countries, are warranted [18, 19].

## Methods

### Objective

This study aimed to identify the determinants of HRQoL among patients with SLE in Hanoi, Vietnam.

### Study design and participants

This cross-sectional study was conducted at the National Hospital of Dermatology and Venereology in Hanoi, Vietnam, in August to September 2019. This hospital is under the Ministry of Health and is one of the largest hospitals in Vietnam. It was chosen as the study site because it provides comprehensive treatments for patients with SLE in Vietnam, where the disease prevalence is unknown.

The inclusion criteria for participants in this study were patients with SLE who were diagnosed with SLE based on the American College of Rheumatology classification and those who were over 18 years old. The exclusion criterion was SLE patients with mental disorders at the time of the study or in the past. Also, patients who stopped coming to the hospital for various reasons were excluded, such as being transferred to other hospitals or passed away.

### Measures

A structured questionnaire (Additional file 1 - Questionnaire) was used to identify the determinants of HRQoL in patients with SLE. This questionnaire consisted of 4 scales: (1) Short Form-36 (SF-36) to assess HRQoL, (2) Multidimensional Scale of Perceived Social Support (MSPSS), (3) Satisfaction with Life Scale (SLS), and (4) Mental Adjustment to SLE. Sociodemographic information (age, gender, education, occupation, marital status, number of children, monthly income, drinking and smoking habits) was also included in the questionnaire. The questionnaire was written in both English and Vietnamese. The Vietnamese version was translated by a competent Vietnamese doctor who was fluent in English, and back translation was performed by an officer of the Ministry of Health, Vietnam, to ensure the accuracy of the translation. A pre-test was conducted among 17 patients with SLE who were randomly recruited from the same hospital prior to data collection to confirm that the questionnaire was understandable to patients with SLE in Vietnam.

The SF-36 has 8 domains and generates 2 summary scores. The physical component summary comprises general health, physical functioning, and role limitations due to physical health and body pain; and the mental component summary consists of role limitations due to emotional problems, social functioning, energy/fatigue, and emotional well-being. For each domain, item scores were coded, summed, and transformed into scores ranging from 0 to 100. A higher score defines a more favorable health state [20–23].

MSPSS measures the social support of family, friends and significant others. The support of each group was measured using a Likert scale from 1 to 7, and the total score was the mean of all 3 groups. The range of the possible score is 1–7. A higher score indicates higher support [8].

The SLS contains 5 questions, and each question was measured using a Likert scale ranging from 1 to 7. The total score ranges from 5 (extremely dissatisfied) to 35 (extremely satisfied) [24].

To measure mental adjustment to SLE, the Mental Adjustment to Cancer Scale was used in this study [25]. The items in the Mental Adjustment to Cancer Scale were reworded for use in patients with SLE. A Japanese SLE expert and the Ethics Committee of the National Hospital of Dermatology and Venereology in Hanoi reviewed and confirmed the face and content validity of this scale. This scale contains 5 domains, measured using a Likert scale from 1 to 4: (1) fighting spirit, (2) helplessness or hopelessness, (3) anxious preoccupation, (4) fatalism, and (5) avoidance. The scale contains questions about how patients with SLE react to the situation of living with SLE as follows: (1) “I try to fight the illness” for fighting spirit, (2) “I feel that life is hopeless” and “I feel completely at a loss about what to do” for helplessness or hopelessness, (3) “I feel fatalistic about it” for fatalism, (4) “I worry about the SLE relapsing or getting worse” for anxious preoccupation, and (5) “I avoid finding out more about it” for avoidance. A higher score for fighting spirit indicates a more positive attitude toward living with SLE. In contrast, a higher score for the remaining 4 domains indicates a more negative attitude toward living with SLE [25, 26].

### Data analysis

Descriptive statistics was used to summarize the sociodemographic data. The mean, standard deviation, and minimum and maximum values were computed for all the exposure variables. Two multiple linear regression models were run to identify the determinants of the physical component summary and mental component summary, controlling for potential confounders. *P* < 0.05 were considered statistically significant. Stata version 15 (StataCorp LLC, College Station, TX, USA) was used for all the analyses.

## Results

The hospital had 670 patients’ medical records at the time of the study. Among these, the medical records of 536 patients were excluded for various reasons. The exact number is not available for each exclusion reason, but some of them were transferred to other hospitals closer to their residences, others had financial problems, lacked understanding for the necessity of treatment, or passed away (personal communication with the medical workers in the hospital.) Even though patients no longer come to the hospital, the hospital must maintain their medical records by following regulations on the storage of medical records. This regulation is issued by the Ministry of Health of Vietnam and must be followed. According to the regulations, inpatient and outpatient medical records are kept for at least 10 years, and the patient’s death record is kept for at least 20 years for educational purposes, such as scientific research. In total, 134 patients were coming to the hospital for regular medical consultation during the study period, and all of them were included in this study as they met the inclusion criteria.

The mean age of all patients was 37.9 years old (SD 12.5). Of 134 patients, 126 (94.0%) were female, and 104 (77.6%) were married (Table 1). Regarding education status, 87 (65.0%) reported having a secondary/tertiary education. Regarding occupation, 61 (45.5%) participants reported having outside work/physical work, while 63 (26.9%) had a skilled work/office work. The majority were nondrinkers (117, 87.3%) and nonsmokers (129, 96.3%).

**Table 1:** Socio-demographic characteristics of patients with SLE (n = 134)

Variable	n	%
**Age**		
(Mean, SD, range)	(37.9, 12.5, 18–66)	
18–24	22	16.4
25–36	45	33.6
37–60	61	45.5
More than 60	6	4.5
**Gender**		
Male	8	6.0
Female	126	94.0
**Education**		
Junior high school and below	47	35.1
High school	53	39.6
University’s degree	34	25.4
**Occupation**		
Outside work/physical work	61	45.5
Skilled work/office work	36	26.9
Others	37	27.6
**Marital status**		
Married	104	77.6
Others	30	22.4
**Children**		
(Mean, range)		(1.5, 0–4)
**Monthly income** (n = 128)		
(Mean, SD) in VND	(7,756,250, 534,541.1)	
(*US$1 = 23,165.27 VND as of 17th November 2019*)		
**Drinking**		
Yes	8	6.0
I ever had, but quit.	9	6.7
Never	117	87.3
**Smoking**		
Yes	2	1.5
I ever had, but quit	3	2.2
Never	129	96.3

Table 2 shows the scores of the mental adjustment, satisfaction with life, and social support. The mean score of satisfaction with life was 23.2 (SD 5.6) which is classified as slightly satisfied. For social support in total, the mean score was 5.8 (SD 1.1). Among the components of social support, the mean score of the social support by family was 6.2 (SD 1.2), which was higher than that of the other components.

**Table 2:** Scores of mental adjustment, satisfaction with life, and social support (n = 134)

Variable	Mean	SD	Min	Max
**Mental adjustment**				
Fighting spirit (16–64)	49.1	6.1	35	64
Helplessness or hopelessness (6–24)	9.9	3.6	6	20
Anxious preoccupation (9–36)	24.4	4.3	15	36
Fatalism (8–32)	19.5	3.0	12	29
Avoidance (1–4)	1.8	0.8	1	4
**Satisfaction with life** (5–35)	23.2	5.6	7	35
**Social support**				
By family (1–7)	6.2	1.2	1	7
By friend (1–7)	5.3	1.4	1	7
By significant other (1–7)	5.9	1.4	1	7
**Total** (1–7)	5.8	1.1	1	7

Table 3 shows the scores of each domain and the physical component and mental component summaries of the SF-36. The mean scores of each summary were 62.3 (SD 19.7) and 65.2 (SD 19.6), respectively.

**Table 3:** Scores of SF-36 domains and component summaries (n = 134)

Variable	Mean	SD	Min	Max
General health (0-100)	47.5	18.7	0	95
Physical functioning (0-100)	73.3	21.2	10	100
Role limitations due to physical health (0-100)	47.6	40.5	0	100
Body pain (0-100)	73.3	28.3	0	100
Role limitations due to emotional problem (0-100)	49.3	42.8	0	100
Social functioning (0-100)	81.5	16.3	37.5	100
Energy/Fatigue (0-100)	64.2	20.7	10	100
Emotional well-being (0-100)	69.1	20.5	16	100
Physical component summary* (0-100)	62.3	19.7	6.9	98.8
Mental component summary ** (0-100)	65.2	19.6	21.1	100

*Physical component summary contains domains of General health, Physical functioning, Role limitations due to physical health, and Bodily pain

**Mental component summary contains domains of Role limitations due to emotional problems, Social functioning, Energy/Fatigue, and Emotional well-being

Table 4 shows the factors associated with the physical and mental component summaries. Older patients were more likely to have a lower mental component summary score (B=-0.45, p = 0.002, 95% CI -0.73, -0.17), but not a physical component summary score. Patients with more children were more likely to have a lower physical component summary score (B=-5.14, p = 0.015, 95% CI -9.27, -1.00). Regarding mental adjustment, patients who felt more helplessness or hopelessness were more likely to have a lower mental component summary score and a lower physical component summary score (B=-1.85, p < 0.001, 95% CI -2.80, -0.90; B=-1.69, p < 0.001, 95% CI -2.57, -0.81). Also, patients who felt more anxious were more likely to have a lower mental component summary score (B=-1.04, p = 0.005, 95% CI -1.77, -0.32). Patients who were more satisfied with their lives have higher physical and mental component summary scores (B = 1.07, p < 0.001, 95% CI 0.50, 1.64; B = 1.08, p < 0.001, 95% CI 0.55, 1.61).

**Table 4:** Factors associated with physical component summary and mental component summary (n = 134)

Variable	Physical component summary				Mental component summary		
	B	*p*	95%CI		B	*p*	95%CI
**Age**	-0.23	0.132	(-0.53 to 0.07)		-0.45	0.002	(-0.73 to -0.17)
**Gender**							
Male	reference				reference		
Female	-4.42	0.456	(-16.12 to 7.28)		-4.28	0.435	(-15.09 to 6.54)
**Education**							
Junior high school and below	reference				reference		
High school	1.68	0.656	(-5.76 to 9.11)		2.29	0.510	(-4.58 to 9.17)
University’s degree	4.18	0.397	(-5.57 to 13.93)		-0.36	0.937	(-9.38 to 8.65)
**Occupation**							
Outside work/physical work	reference				reference		
Skilled work/office work	0.37	0.927	(-7.76 to 8.50)		3.48	0.361	(-4.03 to 11.00)
Others	-3.54	0.325	(-10.64 to 3.56)		-3.40	0.307	(-9.96 to 3.16)
**Marital status**							
Others	reference				reference		
Married	5.03	0.305	(-4.63 to 14.69)		7.88	0.083	(-1.05 to 16.81)
**Children**	-5.14	0.015	(-9.27 to -1.00)		-2.58	0.183	(-6.40 to 1.24)
**Mental adjustment**							
Fighting spirit	-0.04	0.869	(-0.57 to 0.48)		0.12	0.622	(-0.36 to 0.61)
Helplessness or hopelessness	-1.85	< 0.001	(-2.80 to -0.90)		-1.69	< 0.001	(-2.57 to -0.81)
Anxious preoccupation	-0.42	0.293	(-1.20 to 0.37)		-1.04	0.005	(-1.77 to -0.32)
Fatalism	0.52	0.320	(-0.51 to 1.56)		0.48	0.322	(-0.48 to 1.44)
Avoidance	0.38	0.838	(-3.32 to 4.09)		-0.57	0.741	(-4.00 to 2.85)
**Satisfaction with life**	1.07	< 0.001	(0.50 to 1.64)		1.08	< 0.001	(0.55 to 1.61)
Social support							
Low support	reference				reference		
Moderate support	-3.11	0.728	(-20.76 to 14.54)		-10.82	0.192	(-27.14 to 5.49)
High support	-0.33	0.970	(-17.39 to 16.74)		-10.17	0.204	(-25.94 to 5.61)

## Discussion

This study has five main findings. This study was the first to assess the association between mental adjustment and HRQoL among patients with SLE. In this study, patients with SLE who felt more helplessness or hopelessness were more likely to have a worse HRQoL. Also, patients who felt more anxious were more likely to have a lower HRQoL. Moreover, it was found that as patients got older, their HRQoL was more likely to worsen. This study also identified that having more children had a negative association with HRQoL among patients with SLE. Finally, patients with SLE who were more satisfied with their lives were more likely to have a better HRQoL.

Patients with SLE who felt more helplessness or hopelessness were more likely to have a worse HRQoL. Most patients experience common symptoms, such as fatigue and body pain. These symptoms cannot be seen and have been described as “invisible illness” [27]. As a result, these symptoms cause a lack of understanding among their families, friends, and even medical workers [27, 28]. Lack of understanding provokes negative responses from others, such as denying and not acknowledging, which leads to reluctance of patients to tell others about their medical conditions or share their feelings with others [29]. Reluctance to communicate with others can result in feeling helpless or hopeless.

In this study, patients who felt more anxious were more likely to have a lower HRQoL. Patients with SLE in this study received high social support from their families despite feelings of helplessness or hopelessness and anxious preoccupation. Patients who are highly dependent on social support from their family might be concerned that their support would not be continuous for some reasons. Compared to high-income countries, the social context of patients with SLE can influence the differences in the determinants of their HRQoL in lower middle-income countries. For example, previous studies reported the determinants of poor HRQoL in high-income countries were (1) older age, (2) fatigue, and (3) the presence of co-morbid neurological or psychiatric disorders [31]. According to the previous research and this study, the determinants of adverse HRQoL in lower middle-income countries were identified as (1) disease activities, (2) getting old, (3) having more children, (4) emotional and aesthetic concerns, and (5) unemployment [30]. Some of the determinants are similar, but possible social context might cause differences in the determinants of HRQoL in countries with different income levels. For example, adequate medical resources are widely available to detect SLE at an early stage in high-income countries. Furthermore, adequate social security mitigates the effect of SLE on employment and as a result, employment is retained [30, 31]. In contrast, lower middle-income countries lack medical facilities that can detect SLE and have a shortage of medical professionals who can accurately diagnose SLE, both of which can cause differences in the determinants of HRQoL. Patients with SLE also face extra financial burden due to paying for out-of-pocket costs for healthcare [30]. In Vietnam, support from the family plays an important role to compensate for limited welfare services. Moreover, psychiatric disorders such as depression and anxiety are the most commonly described disorders during the disease course [32]. These symptoms accelerate psychological distress, which were also identified as the major predicting factors of impaired HRQoL in other research [32]. Considering the nature of SLE, patients experience uncertainty and anxiety regarding their future.

As patients with SLE aged, their HRQoL was more likely to worsen. Patients with SLE can live a normal life as a result of effective medications that can avert critical conditions and contribute to a higher survival rate. However, some factors can have deleterious effects on patients with SLE, such as controlling chronic symptoms during the disease course, long-term use of medication as well as accumulated disease damage [33, 34]. Also, constant health checkups that cause a financial burden and fears of aggravations or relapse as patients age might negatively affect their HRQoL [35].

Patients with more children were more likely to have a worse HRQoL. This could be due to the child-rearing burden, which resulted in worse HRQoL. Parenting roles such as feeding, bathing, lifting, carrying, and cleaning are physically demanding. The parenting role of the mother is still regarded as one of the most important occupations in women’s lives [36]. More time and labor are consumed to take care of children than to take care of themselves [37, 38]. Moreover, body pain and fatigue are not obvious to other people, but these symptoms cause more difficulties in doing housework and limit full participation in the parenting role of mothers [39]. The physical condition of mothers is an important consideration in parenting [39], especially in conservative cultures, such as Vietnam. Therefore, parenting burden could be a cause of worsening HRQoL.

Patients with SLE who were more satisfied with their lives were more likely to have a better HRQoL. Unpredictable disease courses interfere with their life plans. Important life events cannot continue, such as having a family or a stable job [35]. SLE, a chronic disease, has a great influence on the patients’ HRQoL as well as their satisfaction with life [4]. Fulfillment of patients’ lives by being able to achieve life goals such as raising a family and having a stable job might lead to better HRQoL.

### Strengths and limitations

To the best of our knowledge, this is the first study that assesses mental adjustment and factors associated with HRQoL among patients with SLE. However, this study had several limitations. This was a hospital-based study and might not be representative of the Vietnamese SLE patient population. However, we conducted this study at one of the largest national hospitals in Vietnam, providing comprehensive treatment for SLE patients in Hanoi and other surrounding regions. SLE patients seek diagnosis, treatment, and consultation at this leading hospital. Second, a self-administered questionnaire was used in this research, which could have resulted in desirability bias. However, before data collection, the research assistants explained the purpose of the study and protected confidentiality. Third, patients with mental disorders were excluded based on the medical records, which provided results that did not reflect the situation of patients with mental disorders. Fourth, disease activities and the presence of comorbidities were not included in the analysis due to the incomplete information of the comorbidities in the medical records. Therefore, the results did not consider the disease conditions of the patients. Fifth, instead of age, the disease duration might be more appropriate as an exposure variable in the regression analysis. However, as we did not collect such data, we could not perform a regression analysis on the disease duration in this study. Finally, this study included only out-patients, and did not evaluate the disease severity that might affect their HRQoL.

## Conclusion

Among patients with SLE in Hanoi, Vietnam, factors associated with lower HRQoL were a feeling of helplessness or hopelessness, and burden of parenting roles. However, continuous support can improve the HRQoL of patients with SLE, such as information support to enhance the self-management skills of SLE patients, and family support to alleviate the feeling of helplessness or hopelessness. When resources are limited, self-support groups can provide SLE patients with a safe space to share their anxiety and experiences. At the community level, a school/community center could provide daycare services or after-school activities to reduce the burden of patients’ physical parenting roles.

## Electronic supplementary material

Below is the link to the electronic supplementary material.

Supplementary Material 1: Questionnaire

## Data Availability

The datasets analyzed are presented in the main manuscript.

## Abbreviations

SLE: Systemic lupus erythematosus
HRQoL: Health-related quality of life
PCS: Physical component summary
MCS: Mental component summary
SF-36: Short Form-36
MSPSS: Multidimensional Scale of Perceived Social Support
SLS: Satisfaction with Life Scale
