# Supplementary material for: Determinants of health-related quality of life among patients with systemic lupus erythematosus in Hanoi, Vietnam
Source: BMC Rheumatol. 2023 Jun 22;7:16. doi: 10.1186/s41927-023-00339-6 (PMC10286429; doi:10.1186/s41927-023-00339-6)
Supplement: Supplementary file 1 — Supplementary Material 1: Questionnaire [file 41927_2023_339_MOESM1_ESM.docx]

**Questionnaire (English Version)**

**The association of mental adjustment, social support and health related quality of life among people living with systemic lupus erythematosus in Hanoi, Vietnam.**

**Number of Questionnaire □□□**

**Date:**

**Please answer the questions of the Health Survey completely and, honestly , preferably in a quiet place where you will not be interrupted.**

**Section 1: Socio-demographic Information**

1. How old are you?

years old

1. What is your gender?
2. Male
3. Female
4. What is your marital status?
5. Single (no relationship)
6. Single (in relationship)
7. Married
8. Divorced/widowed
9. How many children do you have?

1. What is your highest education level?
2. Elementary school
3. Junior high school
4. High school
5. Bachelor’s degree
6. Master’s degree
7. Doctoral degree
8. What is your occupation?
9. Office work (staying inside of building)
10. Outside work (for example, agriculture, selling foods at vendor stall)
11. Heavy physical work
12. Skilled work
13. Housewives
14. Jobless
15. Others
16. How much is your monthly average household income?

1. Do you smoke?
2. Yes
3. I ever had, but quit
4. Never
5. Do you drink?
6. Yes
7. I ever had, but quit
8. Never
9. Is there anything you do in daily life to prevent worse situations of your disease?

**Section 2 : Quality of Life**

1. In general, would you say your health is:
2. Excellent
3. Very Good
4. Good
5. Fair
6. Poor
7. Compared to one year ago, how would you rate your health in general now?
8. Much better now than one year ago
9. Somewhat better now than one year ago
10. About the same
11. Somewhat worse now than one year ago
12. Much worse than one year ago

The following items are about activities you might do during a typical day.

Does your health now limit you in these activities? If so, how much?

|  | | Yes, limited a lot. | Yes, limited a little | No, not limited at all |
| --- | --- | --- | --- | --- |
| 3. | Vigorous activities, such as running, lifting heavy objects, participating in strenuous sports. |  |  |  |
| 4. | Moderate activities, such as moving a table, pushing a vacuum cleaner, bowling, or playing golf. |  |  |  |
| 5. | Lifting or carrying groceries. |  |  |  |
| 6. | Climbing several flights of stairs. |  |  |  |
| 7. | Climbing one flight of stairs. |  |  |  |
| 8. | Bending, kneeling, or stooping. |  |  |  |
| 9. | Walking more than a mile. |  |  |  |
| 10. | Walking several blocks. |  |  |  |
| 11. | Walking one block. |  |  |  |
| 12. | Bathing or dressing yourself. |  |  |  |

During the past 4 weeks, have you had any of the following problems with your work or other regular daily activities as a result of your physical health?

|  | | Yes | No |
| --- | --- | --- | --- |
| 13. | Cut down the amount of time you spent on work or other activities. |  |  |
| 14. | Accomplished less than you would like. |  |  |
| 15. | Were limited in the kind of work or other activities. |  |  |
| 16. | Had difficulty performing the work of other activities (for example, it took extra effort). |  |  |

During the past 4 weeks, have you had any of the following problems with your work or other regular daily activities as a result of any emotional problems (such as feeling depressed or anxious)?

|  | | Yes | No |
| --- | --- | --- | --- |
| 17. | Cut down the amount of time you spent on work or other activities. |  |  |
| 18. | Accomplished less than you would like. |  |  |
| 19. | Didn’t go work or other activities as carefully as usual. |  |  |

20. Emotional problems interfered with your normal social activities with family, friends, neighbors, or groups?

a. Not at all

b. Slightly

c. Moderately

d. Severe

e. Very severe

21. How much bodily pain have you had during the past 4 weeks?

a. None

b. Very mild

c. Mild

d. Moderate

e. Severe

f. Very severe

22. During the past 4 weeks, how much did pain interfere with your normal work (including both work outside the home and housework)

a. Not at all

b. A little bit

c. Moderately

d. Quite a bit

e. Extremely

These questions are about how you feel and how things have been with you during the last 4 weeks. For each questions, please give the answer that comes closest to the way you have been feeling.

|  | | All of the time | Most of the time | A good bit of the time | Some of the time | A little bit of the time | None of the time |
| --- | --- | --- | --- | --- | --- | --- | --- |
| 23. | Did you feel full of pep? |  |  |  |  |  |  |
| 24. | Have you been a very nervous person? |  |  |  |  |  |  |
| 25. | Have you felt so down in the dumps that nothing could cheer you up? |  |  |  |  |  |  |
| 26. | Have you felt calm and peaceful? |  |  |  |  |  |  |
| 27. | Did you have a lot of energy? |  |  |  |  |  |  |
| 28. | Have you felt downhearted and blue? |  |  |  |  |  |  |
| 29. | Did you feel worn out? |  |  |  |  |  |  |
| 30. | Have you been a happy person? |  |  |  |  |  |  |
| 31. | Did you feel tired? |  |  |  |  |  |  |

32. During the past 4 weeks, how much of the time has your physical health or emotional problems interfered with your social activities (like visiting with friends, relatives, etc.)?

a. All of the time

b. Most of the time

c. Some of the time

d. A little of the time

e. None of the time

How true or false is each of the following statements for you?

|  | | Definitely true | Mostly true | Don’t know | Mostly false | Definitely false |
| --- | --- | --- | --- | --- | --- | --- |
| 33. | I seem to get sick a little easier than other people. |  |  |  |  |  |
| 34. | I am as healthy as anybody I know. |  |  |  |  |  |
| 35. | I expect my health to get worse. |  |  |  |  |  |
| 36. | My health is excellent. |  |  |  |  |  |

**Section 3: Social Support**

Instructions: We are interested in how you feel about the following statements.

Read each statement carefully. Indicate how you feel about each statement.

Check the box of number “1” if you Very Strongly Disagree

Check the box of number “2” if you Strongly Disagree

Check the box of number “3” if you Mildly Disagree

Check the box of number “4” if you are Neutral

Check the box of number “5” if you Mildly Agree

Check the box of number “6” if you Strongly Agree

Check the box of number “7” if you Very Strongly Agree

|  | | 1 | 2 | 3 | 4 | 5 | 6 | 7 |
| --- | --- | --- | --- | --- | --- | --- | --- | --- |
| 1. | There is a special person who is around when I am in need. |  |  |  |  |  |  |  |
| 2. | There is a special person with whom I can share my joys and sorrows. |  |  |  |  |  |  |  |
| 3. | My family really tries to help me. |  |  |  |  |  |  |  |
| 4. | I get the emotional help and support I need from my family. |  |  |  |  |  |  |  |
| 5. | I have a special person who is a real source of comfort me. |  |  |  |  |  |  |  |
| 6. | My friends really try to help me. |  |  |  |  |  |  |  |
| 7. | I can count on my friends when things go wrong. |  |  |  |  |  |  |  |
| 8. | I can talk about my problems with my family. |  |  |  |  |  |  |  |
| 9. | I have friends with whom I can share my joys and sorrows. |  |  |  |  |  |  |  |
| 10. | There is a special person in my life who cares about my feeling. |  |  |  |  |  |  |  |
| 11. | My family is willing to help me make decisions. |  |  |  |  |  |  |  |
| 12. | I can talk about my problems with my friends. |  |  |  |  |  |  |  |

**Section 4: Mental Adjustment**

A number of statements are given below which describe people’s reactions to having SLE. Please check the box of the appropriate number to the right of each statement, indicating how far it applies to you at present.

Check the box of number “1” if it Definitely does not apply to you

Check the box of number “2” if it does not apply to you

Check the box of number “3” if it apply to you

Check the box of number “4” if it Definitely apply to you

|  | | 1 | 2 | 3 | 4 |
| --- | --- | --- | --- | --- | --- |
| 1. | I keep quite busy, so I don’t have time to think about it. |  |  |  |  |
| 2. | Other people worry about me more than I do. |  |  |  |  |
| 3. | I feel that my positive attitude will benefit my health. |  |  |  |  |
| 4. | I try to fight the illness. |  |  |  |  |
| 5. | I am determined to put it all behind me. |  |  |  |  |
| 6. | Since diagnosed as SLE, I now realize how precious life is, and I’m making the most of it. |  |  |  |  |
| 7. | I try to have a very positive attitude. |  |  |  |  |
| 8. | I think of other people who are worse off. |  |  |  |  |
| 9. | I firmly believe that I will get better. |  |  |  |  |
| 10. | I see my illness as a challenge. |  |  |  |  |
| 11. | I try to keep a sense of humor about it. |  |  |  |  |
| 12. | I have plants for the future, eg, holiday, jobs, housing. |  |  |  |  |
| 13. | I count my blessing. |  |  |  |  |
| 14. | I don’t dwell on my illness. |  |  |  |  |
| 15. | I think my state of mind can make a lot of difference to my health. |  |  |  |  |
| 16. | I try to carry on my life as I’ve always done. |  |  |  |  |

|  | | 1 | 2 | 3 | 4 |
| --- | --- | --- | --- | --- | --- |
| 17. | I feel like giving up. |  |  |  |  |
| 18. | I feel that life is hopeless. |  |  |  |  |
| 19. | I feel completely at a loss about what to do. |  |  |  |  |
| 20. | I am not very hopeful about the future. |  |  |  |  |
| 21. | I feel that there is nothing I can do to help myself. |  |  |  |  |
| 22. | I feel I can’t do anything to cheer myself up. |  |  |  |  |

|  | | 1 | 2 | 3 | 4 |
| --- | --- | --- | --- | --- | --- |
| 23. | I would like to make contact with others in the same boat. |  |  |  |  |
| 24. | I am trying to get as much information as I can about SLE. |  |  |  |  |
| 25. | I feel that problems with my health prevent me from planning ahead. |  |  |  |  |
| 26. | I feel very angry about what has happened. |  |  |  |  |
| 27. | I suffer great anxiety about it. |  |  |  |  |
| 28. | I worry about the SLE relapsing or getting worse. |  |  |  |  |
| 29. | I have difficulty in believing this has happened to me. |  |  |  |  |
| 30. | I have been doing things that I believe will improve my health, eg, exercise. |  |  |  |  |
| 31. | I have been doing things that I believe will improve my health, eg, changed my diet. |  |  |  |  |

|  | | 1 | 2 | 3 | 4 |
| --- | --- | --- | --- | --- | --- |
| 32. | At the moment I take 1 day at a time. |  |  |  |  |
| 33. | I’ve put myself in the hands of God. |  |  |  |  |
| 34. | I feel fatalistic about it. |  |  |  |  |
| 35. | I feel that nothing I can do will make any difference. |  |  |  |  |
| 36. | I’ve had a good life and what’s left is bonus. |  |  |  |  |
| 37. | I’ve left it all to my doctors. |  |  |  |  |
| 38. | I feel that I can’t control what is happening. |  |  |  |  |
| 39. | I avoid finding out more about it. |  |  |  |  |

|  | | 1 | 2 | 3 | 4 |
| --- | --- | --- | --- | --- | --- |
| 40. | I don’t really believe I have SLE. |  |  |  |  |

**Section 5: Satisfaction with Life**

Below are five statements that you may agree or disagree with. Indicate your agreement.

|  | | Strongly disagree | Disagree | Slightly disagree | Neither agree nor disagree | Slightly agree | Agree | Strongly agree |
| --- | --- | --- | --- | --- | --- | --- | --- | --- |
| 1. | In most ways my life is close to my ideal. |  |  |  |  |  |  |  |
| 2. | The conditions of my life are excellent. |  |  |  |  |  |  |  |
| 3. | I am satisfied with my life. |  |  |  |  |  |  |  |
| 4. | So far I have gotten the important things I want in life. |  |  |  |  |  |  |  |
| 5. | If I could live my life over, I would change almost nothing. |  |  |  |  |  |  |  |

**Section 6: Access to hospital**

1. How long does it take from your residence of the hospital?
2. Less than 30 minutes
3. 30-60 minutes
4. More than 1 hour
5. More than 90 minutes
6. More than 2 hour
7. Means of transportation
8. On foot
9. Using public transportation such as bus
10. Using motorcycle
11. Using your own car
12. Using bicycle
13. When coming to hospital, you come alone or have accompanies?
14. Coming alone always
15. Having accompanies

If you chose “having accompanies”, how often do you have them?

1. Every time (100% of your visiting)
2. Often (about 60% of your visiting)
3. Sometimes (about 50% of your visiting)
4. Less than above

What is your relationship to the person accompanying you?

1. Spouse
2. Other family members
3. Friends
4. Co-workers
5. Children

**Section 7: Medical records**

1. What age when diagnosed?

years old

1. What kind of medications and how many of them do you take?
2. Prednisolone mg
3. Immune suppressors tablets
4. Others
5. What are your main symptoms?
